# Forensic Perspectives on Child Sexual Abuse Disclosure in Greece: A Retrospective Study

**DOI:** 10.3390/pediatric18010012

**Published:** 2026-01-16

**Authors:** Konstantinos Dimitriou, Vasiliki Efthymiou, Kallirroi Fragkou, Pierre-Antoine Peyron, Laurent Martrille, Eric Baccino, Flora Bacopoulou, Stavroula Papadodima

**Affiliations:** 1 Department of Forensic Medicine and Toxicology, School of Medicine, National and Kapodistrian University of Athens, 115 27 Athens, Greece; kondi@med.uoa.gr (K.D.); roifragou@gmail.com (K.F.); 2 Center for Adolescent Medicine and UNESCO Chair in Adolescence Health Care, First Department of Pediatrics, School of Medicine, National and Kapodistrian University of Athens, Aghia Sophia Children’s Hospital, 115 27 Athens, Greece; vikimiou2003@yahoo.gr (V.E.); fbacopoulou@med.uoa.gr (F.B.); 3 Institute of Forensic Medicine, CHU Reunion, University of Reunion, 97400 Reunion, France; pierre-antoine.peyron@chu-reunion.fr; 4 EDPFM, Department of Forensic Medicine CHU Montpellier, University of Montpellier, 34000 Montpellier, France; laurent.martrille@chu-montpellier.fr (L.M.); e-baccino@chu-montpellier.fr (E.B.)

**Keywords:** child sexual abuse, disclosure, forensic medicine, Greece, medico-legal examination, anogenital findings, disclosure, Unités d’Accueil Pédiatrique Enfant en Danger (UAPED)

## Abstract

**Purpose:** Child sexual abuse (CSA) is a major public health and forensic concern, often involving delayed disclosure that limits evidence collection and affects judicial outcomes. This study analyzed disclosure patterns, victim–perpetrator characteristics, and forensic findings in CSA cases evaluated in Greece, contributing to the limited Southern European evidence base. **Material and Methods:** A retrospective review of 89 CSA cases (2014–2024) examined by a certified forensic physician at the Department of Forensic Medicine and Toxicology, National and Kapodistrian University of Athens, was conducted. Data from official medico-legal reports included demographics, abuse context, forensic findings, and disclosure interval. Statistical analyses explored factors associated with delayed disclosure (>7 days). **Results:** Victims were predominantly female (69.7%) with a mean age of 9.8 years. Most perpetrators were adult males, and over half of cases (53.9%) involved intrafamilial abuse. The mean delay in disclosure was 79 days; only 29.2% reported within one week. Recurrent abuse correlated with delayed disclosure (*p* = 0.006), while early disclosure was associated with biological evidence collection (*p* < 0.001). Physical injuries were observed in 23.6% of victims, genital findings in 17%, and anal findings in 3.4%. **Conclusions:** Delayed disclosure was common and significantly reduced the likelihood of identifying forensic evidence. The early application of trauma-informed examinations, which adopt a child-centered approach emphasizing safety, emotional regulation, and the prevention of re-traumatization, is essential for the medical and forensic evaluation of abused children. Adopting hospital-based multidisciplinary units could improve forensic documentation, interagency coordination, and psychosocial care in Greece.

## 1. Introduction

Child sexual abuse (CSA) represents one of the most pervasive and devastating forms of violence against children, with profound and enduring consequences for physical, psychological, and social well-being [1,2]. Despite growing awareness and the implementation of child protection frameworks, recent global evidence shows that CSA remains a major public health concern, with an estimated lifetime prevalence of 11% for sexual harassment, 9% for contact sexual violence, and 6% for forced sexual intercourse (7% among girls and 3% among boys) [3]. Yet, the vast majority of cases remain undetected or unreported, primarily due to the complex dynamics surrounding disclosure [4,5].

Disclosure of CSA is not a single event but a gradual and emotionally charged process shaped by multiple interacting factors. These include the child’s age and developmental stage, the relationship to the perpetrator, the nature, frequency, and severity of the abuse, and the availability of supportive adults [6,7,8]. Evidence consistently shows that younger age and intrafamilial perpetration are the strongest predictors of delayed or absent disclosure [6,7,8,9,10]. Cultural norms emphasizing family loyalty, modesty, and social reputation further reinforce silence, particularly in collectivist societies [11,12].

From a forensic standpoint, the timing of disclosure has direct implications for the detection of injuries and biological evidence. The likelihood of identifying acute genital or anal trauma or retrieving viable DNA traces declines sharply after the first 72 h following the assault [13,14,15,16]. Consequently, most examinations, especially those conducted after delayed reporting, yield normal or nonspecific findings, underscoring that the absence of physical evidence does not equate to the absence of abuse. Delayed reporting has also been shown to affect the criminal justice outcome of CSA cases by reducing the availability of corroborative evidence and decreasing the likelihood that cases proceed to prosecution [17]. Importantly, this impact is not uniform: delays may influence legal decisions differently across victim subgroups, as demonstrated by Bunting, who reported variations depending on age and sex [18].

This evidentiary limitation elevates the importance of trauma-informed examination, contextual analysis, and multidisciplinary collaboration in CSA investigations [19,20]. A trauma-informed examination is an evaluative approach that recognizes the psychological and physiological impact of trauma and adapts forensic and medical procedures to promote safety, predictability, and emotional regulation. It emphasizes clear communication, supportive interactions, and respect for the child’s pace and autonomy, with the goal of minimizing distress and preventing re-traumatization [21,22]. Although international research has increasingly addressed CSA, data from Southern and Southeastern Europe remain relatively sparse. Sociocultural and systemic factors in these regions may shape patterns of disclosure and influence the forensic management of CSA cases. The aim of the present study is to contribute to this limited body of evidence by examining demographic, contextual, and disclosure characteristics of CSA cases referred for forensic evaluation in Greece. It further explores factors associated with delayed disclosure and their forensic implications, offering empirical observations that may inform context-sensitive, child-centered investigative approaches.

## 2. Materials and Methods

### 2.1. Study Design and Setting

This was a retrospective observational study based on medico-legal records of child sexual abuse (CSA) cases examined between January 2014 and December 2024. The study sample consisted of consecutive forensic examinations conducted by a single certified forensic doctor (S.P.) at the Department of Forensic Medicine and Toxicology, National and Kapodistrian University of Athens.

In Greece, the forensic examination of minors in cases of sexual abuse is conducted exclusively upon an official referral order issued by the Investigative Authorities (Judicial Authorities and Police). Examinations are typically performed immediately after referral, particularly when the incident involves a recent event rather than past abuse, to ensure timely clinical assessment and evidence collection.

These cases did not represent the department’s total case volume but constituted a complete consecutive series for that examiner, thereby ensuring consistency in examination procedures and documentation practices. Although each forensic service covers a relatively stable geographic area, minor fluctuations in internal case allocation (e.g., during staff leave or training periods) may occur, reflecting routine operational factors rather than examiner-driven selection. As our analyses focus on associations within the referred cases, rather than temporal incidence trends, such variability does not materially affect the internal validity of the correlations reported.

### 2.2. Forensic Examination

In Greece, forensic examinations cases are regularly performed by a single appointed forensic specialist nationwide. Only in university departments may a resident be present for training purposes In our Department, all examinations are performed by a two-physician team consisting of one certified forensic specialist and one resident in forensic medicine. Both physicians were present during the clinical assessment, contributed to the documentation of findings, and participated in drafting the medico-legal report, which is formally signed by the specialist in accordance with Greek legislation.

All assessments were performed with efforts to ensure comfort, privacy, and minimization of any additional distress. When acute symptoms were present, when hospital-based equipment was required, or when pediatric co-assessment was deemed clinically beneficial—the examination took place in a hospital setting in collaboration with pediatricians.

The clinical examination included a general physical assessment, inspection of the skin, and anogenital and anal examination using adequate illumination and non-invasive techniques appropriate for minors. No speculum examination was performed in prepubertal children. Findings were documented in detail in the medico-legal report using standard anatomical terminology.

Biological samples for DNA analysis were collected in accordance with current national forensic practice. Swabs from the genital area were obtained when alleged vaginal contact or penetration had occurred within approximately six days, and from the anal area when the reported event had taken place within two to three days. When the examination occurred on the same day as disclosure and the child had not bathed, additional swabs from other body sites (e.g., oral cavity, neck, or skin surfaces reported as having been licked or kissed) were collected when indicated. Underwear, clothing, and bed linen provided by the Investigative Authorities were also preserved for laboratory analysis. In Greece the laboratory analysis of all swabs is conducted exclusively by the Investigative Authorities. The forensic services do not receive the analytical results, and therefore these outcomes could not be included in the present study.

Colposcopy and systematic photo- or video-documentation of anogenital findings are not routinely available in our service and are not mandated by Greek legislation in cases of child sexual abuse. Consequently, anogenital lesions were recorded primarily through detailed written descriptions rather than colposcopic imaging.

Microbiological testing for sexually transmitted infections was not performed systematically but requested selectively, depending on the child’s age, the type and timing of reported contact, clinical symptoms, and case-specific risk factors. Such tests were conducted in collaboration with hospital laboratories, acknowledging that the availability of certain assays—such as Nucleic Acid Amplification Tests (NAATs) for *Chlamydia trachomatis*—remains limited within the Greek public health system.

### 2.3. Participants and Inclusion and Exclusion Criteria

Cases were eligible for inclusion if they met all of the following criteria:

- The examinee was a minor (<18 years old) at the time of the forensic examination.
- The case concerned alleged sexual abuse, defined as any act of sexual contact, coercion, penetration, or exploitation as documented in the official referral by the Investigative Authorities.
- The child underwent a full forensic examination conducted at the Department of Forensic Medicine and Toxicology, National and Kapodistrian University of Athens.
- A complete official medico-legal report was available, allowing extraction of demographic, contextual, clinical, and procedural variables.

Cases were excluded if any of the following applied:

- Allegations not concerning sexual abuse, such as physical abuse only, neglect, or non-sexual maltreatment.
- Cases without a forensic clinical examination, including those where the child was referred but did not present, or the examination was cancelled.
- Duplicate referrals, in which only the first complete forensic evaluation was retained.

Finally, eighty-nine minors (<18 years) referred by the Investigative Authorities for forensic examination following allegations of sexual abuse were included.

### 2.4. Data Collection and Variables

Data were extracted from official forensic reports and accompanying documents (e.g., police referrals, pediatric or psychological evaluations, and laboratory submissions). Data extraction was performed independently by two reviewers (one certified forensic physician-K.D. and one resident in Forensic Medicine with 3.5 years of experience-K.F.), and discrepancies were resolved by consensus.

The following variables were analyzed:

- Victim characteristics: age, sex, nationality, and coexisting health conditions.
- Perpetrator characteristics: age group (adult/minor), sex, nationality, and relationship to the victim (intrafamilial or extrafamilial).
- Contextual variables: place of occurrence, number of perpetrators, frequency of abuse, and type of sexual act (touching, intercourse, etc.).
- Forensic findings: presence of physical injuries, genital/anal findings, performance of toxicological analysis and toxicological results, and collection of biological material.
- Procedural variables: involvement of other medical specialties and time interval between abuse onset and disclosure to authorities.

Physical injuries refer to findings on the body surface and exclude genital or anal findings, which were documented as separate categories. They were documented based on findings observed during the forensic examination. When available, medical records from prior pediatric or emergency assessments were reviewed to corroborate or contextualize injuries.

The primary outcome was the delay in disclosure, defined as the number of days between the reported assault and the day of the first forensic medical consultation. Disclosure delay was categorized as: within 7 days, 8–30 days, and >30 days. Ιn cases reporting multiple episodes of abuse, the disclosure interval was calculated using the most recent alleged incident. Information regarding earlier episodes was frequently incomplete or difficult for children to recall with precision, a well-recognized challenge in forensic evaluations of minors. Because reliable chronological data for initial or remote episodes were often unavailable, the most recent episode provided the only consistently documented and analytically comparable time point across cases.

### 2.5. Statistical Analysis

Descriptive statistics were calculated for all variables. Categorical data are presented as frequencies and percentages, and continuous variables as mean ± standard deviation (SD). Associations between disclosure delay and categorical predictors were assessed using chi-square or Monte Carlo tests, while continuous variables were analyzed using one-way Analysis of Variance ANOVA. Variables with *p* < 0.10 in univariate analysis were included in a multivariate logistic regression model to identify independent predictors of delayed disclosure (>7 days). The cut-off of >7 days was selected based on forensic and clinical considerations. In pediatric sexual abuse, acute anogenital injuries and recoverable biological traces are most likely to be identified within the first days following the assault, while rapid mucosal healing and evidence loss significantly reduce detection rates beyond this period, a threshold that has also been commonly adopted in previous studies (Wood, Lanthier). All analyses were performed using IBM SPSS Statistics v.29.0 (IBM Corp., Armonk, NY, USA), with statistical significance set at *p* < 0.05.

### 2.6. Ethical Considerations

Formal approval by an institutional ethics committee was not required, as the study involved a retrospective analysis of anonymized medico-legal case files obtained under judicial mandate. No contact with victims or families occurred, and all personal identifiers were removed prior to analysis.

The study adhered to the principles of the Declaration of Helsinki and complied with national data protection regulations governing the research use of forensic records. Data were collected in compliance with the General Data Protection Regulation (GDPR) (EU) 2016/679.

## 3. Results

Table 1 presents the descriptive statistics of the study variables for the total sample (N = 89). The mean age of the victims was 9.84 years (SD = 4.53). The majority of cases were recorded between 2020 and 2024 (58.4%), with the remainder occurring from 2014 to 2019 (41.6%).

Most victims were of Greek nationality (74.2%), and 69.7% were female. Regarding the preparator, 31.5% were adults, 13.5% were minors, and in 55% of cases, age data was not available. The perpetrators were predominantly male (86.5%), while 2.2% were female, and 3.4% identified as other; data were missing in 7.8% of cases. In terms of nationality, 56.2% of preparators were Greek, 14.6% were of other nationalities, and for 29.2%, this information was not available.

The relationship between the victim and the perpetrators was intrafamilial in over half the cases (53.9%), with biological parents being the most commonly identified perpetrators (40.4%). Extra-familial relationships accounted for 20.2% of cases, while 25.9% had missing data on this variable. The abuse most frequently occurred at home (49.4%), followed by other locations (25.8%) and schools (5.6%). Regarding frequency, in 37.1% of cases, the abuse occurred more than five times, while a single perpetrator was involved in the majority of incidents (75.3%).

Toxicological examination was performed in 6.8% of the cases, and in all of them, no substances were detected. The reported sexual act most involved sexual contact or touching (53.9%) and intercourse (41.6%). In terms of the means used, the penis was involved in 22.5% of cases, hands/fingers in 18%, and multiple means in 5.6%, though data were missing in over half the cases (53.9%).

Physical injuries were documented in 23.6% of the victims. Genital abnormalities were identified in 15 examinations (17%). These included acute or healed hymenal tears in 14 cases and anogenital warts in one case. Anal abnormalities were observed in three children (3.4%), consisting of anogenital warts in two cases and mild anal erythema with superficial mucosal disruption in one case. Co-occurring health issues were identified in 15.4% of victims.

In 65.1% of the cases, other medical specialties (i.e., pediatricians) were involved in the evaluation. The mean time interval between abuse onset and first disclosure to authority was 79.41 days (SD = 130.79). Only 29.2% of victims were examined within the first 7 days after the incident. Lastly, genetic material was collected in 17.4% of cases.

Table 2 presents the crosstabulation between the time taken to disclose abuse and various study variables among 89 cases. No statistically significant associations were found between disclosure time and the disclosure’s year (*p* = 0.356), victim’s nationality (*p* = 0.196), or sex (*p* = 0.600). Similarly, no significant relationship was observed with the perpetrator’s age (*p* = 0.515), nationality (*p* = 0.504), or gender (*p* > 0.999).

Although not statistically significant, intrafamilial abuse was more frequent in cases where disclosure occurred after more than 30 days (91.7%) compared to earlier disclosures (*p* = 0.099). The location of the incident did not significantly affect disclosure time (*p* = 0.214), though incidents occurring at home were more common in delayed disclosures (>30 days: 69.2%).

A significant association was found between the number of abuse occurrences and the time taken to disclose (*p* = 0.006). Victims who disclosed after more than 8 days were more likely to report repeated abuse (>5 times: 78.6% for 8–30 days, 69.2% for >30 days), whereas earlier disclosures more frequently involved single incidents (65.0%).

The involvement of other medical specialties was significantly associated with delayed disclosure (*p* = 0.047), with a higher proportion of such involvement in cases disclosed after 8 days. Specifically, in cases where disclosure was delayed (8–30 days and >30 days), involvement of other specialties was reported more frequently (73.3% and 68.8%, respectively), compared to cases disclosed within 7 days (38.5%).

Additionally, genetic material collection was strongly associated with earlier disclosure (*p* < 0.001), present in 52.0% of cases disclosed within 7 days and absent in all cases disclosed after 30 days.

No significant associations were found between disclosure time and variables such as number of perpetrators (*p* = 0.446), performance of toxicological analysis (*p* = 0.439), type of act (*p* = 0.651), means of perpetration (*p* = 0.290), physical injuries (*p* = 0.922), findings from the genitals (*p* = 0.715), or victim’s pre-existing health problems (*p* = 0.583).

Moreover, a logistic regression was conducted with the time taken to disclose as the dependent variable and the number of child abuse incidents, the involvement of another specialty, and the collection of genetic material as the independent variables. The resulting logistic model had Nagelkerke R^2^ = 0.551, Hosmer–Lemeshow goodness-of-fit test equal to 0.897 with *p* = 0.970. The only significant variable was the number of child abuse incidents (>5), with an OR = 6.803 (95% CI: 1.182–39.150).

## 4. Discussion

This study contributes to the limited forensic evidence base on child sexual abuse (CSA) in Greece, highlighting key patterns of disclosure, victim and perpetrator characteristics, and their implications for forensic practice. Delayed disclosure emerged as a frequent finding, with a mean delay of approximately 79 days and only 29% of victims revealed the abuse within the first week. These findings align with international research demonstrating that delayed reporting of CSA is a pervasive global challenge shaped by psychological, familial, and cultural dynamics [7,8,9,10].

Most victims in this Greek series were female (70%) with a mean age of 9.8 years, a distribution consistent with large-scale meta-analyses confirming that girls are affected approximately twice as often as boys [4,23]. Similar demographic profiles have been reported across Europe, where victim ages typically cluster in late childhood and early adolescence. In Spain, 89% of victims were female with the majority (59%) aged 13–17 years, mean age 11.8 [24], while in Ireland most victims examined at paediatric forensic centres were girls aged 5–11 years [25]. Comparable demographic patterns were also observed in Turkey, where adolescent girls constituted the majority of victims [26] and in Hong Kong, where the median age was 13 years in a large cohort [27].

The majority of perpetrators in this series were adult males, and more than half of the assaults occurred within the family, most commonly involving fathers, stepfathers, or other close relatives. This distribution is consistent with international data indicating that CSA often occurs within environments of trust or dependence [28,29]. Studies from Spain [24], Portugal [30], and France [31] have also highlighted that a substantial proportion of child sexual abuse cases are intrafamilial or involve known perpetrators, particularly among younger victims, where such relationships contribute to delayed disclosure. Although intrafamilial abuse has been widely associated with delayed disclosure in the literature, this relationship did not reach statistical significance in our sample and should therefore be interpreted cautiously.

Genital abnormalities were identified in 15 examinations (17% of cases). Of these, 14 involved acute or healed hymenal tears or disruptions, findings that are classified in contemporary forensic pediatric guidelines as specific or highly suggestive of sexual abuse. The remaining case involved anogenital warts in an 11-year-old girl. Although anogenital warts are generally considered a non-specific finding in prepubertal children, their occurrence at this age is clinically concerning and may raise suspicion of sexual transmission. Anal abnormalities were documented in three cases (3.4%): anogenital warts in two children (an 11-year-old girl and a 3-year-old boy) and mild anal erythema with superficial mucosal disruption in one child. All three findings are classified as non-specific in the pediatric forensic literature, although, as noted above, anogenital warts in an 11-year-old merit careful consideration due to the increased likelihood of sexual transmission in this age group [13,15].

These relatively low detection rates regarding forensic examination are consistent with international research, which shows that most examinations yield normal or non-specific results, particularly when performed outside the acute period [13,32,33]. The frequency of anogenital findings in our cohort (17% genital; 3.4% anal) is consistent with the broader European forensic literature, which repeatedly demonstrates that most examinations in suspected child sexual abuse (CSA) show normal or non-specific findings. Recent retrospective studies from Ireland and Spain report diagnostic or highly suggestive lesions in fewer than 10% of cases, reflecting the effects of delayed disclosure, rapid mucosal healing, and the predominance of non-penetrative acts [25,34]. Earlier European work similarly highlights the limited sensitivity of anogenital examination: Herrmann et al. (2014) showed that over 90% of confirmed CSA victims present with no specific physical abnormalities [35]. In Italy, more than 80% of findings were classified as normal or aspecific under the Adams (2001) system [36]. Comparable low detection rates have been reported in Eastern Europe, including a recent Bulgarian forensic cohort [37].

The likelihood of identifying physical or biological evidence is strongly time-dependent, with substantially higher recovery rates in acute examinations and a marked decline thereafter [38]. In prepubertal victims, clothing and bedding often provide more probative evidence than body swabs collected after 24–72 h [13,39]. Although DNA samples were obtained when clinically and forensically indicated, the outcomes of the laboratory analyses were not accessible to the forensic service, as such results are retained by the Investigative Authorities. Consequently, the study could not examine the relationship between time since the assault and the likelihood of positive DNA findings, a factor known to influence evidence recovery in child sexual abuse cases. Regarding physical injuries, it should be noted that the prevalence of physical injuries reported in this study reflects only those findings that were detectable at the time of examination or described in medical records. Given that medical documentation from prior healthcare encounters was not available for all cases, and considering the rapid healing of pediatric tissues, some injuries may have resolved before the forensic assessment, particularly in cases with delayed disclosure.

Several factors contribute to the absence of positive findings, including the frequent occurrence of non-penetrative sexual acts, the rapid healing of anogenital mucosa in children, and the loss or degradation of biological material due to bathing, clothing changes, or condom use [13,39]. Importantly, a normal anogenital examination does not preclude sexual abuse, as the absence of findings may result from non-penetrative contact, delayed disclosure, or complete tissue healing by the time of examination. Current clinical and forensic guidelines therefore emphasize that the child’s disclosure, behavioral indicators, and psychosocial context remain central to diagnostic interpretation [13].

It should also be noted that, while the majority of allegations are made in good faith, the literature indicates that intentionally false reports are uncommon. However, studies suggest that such allegations may arise more frequently in the context of parental separation or custody disputes, underscoring the importance of multidisciplinary assessment and objective documentation [40,41].

The types of acts reported—predominantly contact forms such as touching or fondling (53.9%) and, less frequently, digital, penile, or anal penetration (41.6%)—closely resemble patterns described in European forensic series. Across studies from Spain [24], Portugal [30], Ireland [25], non-penetrative sexual acts generally account for approximately 55–75% of reported cases, whereas penetrative abuse represents about 25–45%.

Recurrent abuse was identified in 37.1% of cases, confirming that chronic victimization is a defining feature of CSA, particularly in intrafamilial contexts where secrecy, emotional dependence, and fear of disclosure impede early detection [11,28,29]. Overall, the findings of this study align with the broader European and international literature, underscoring the cross-cultural stability of CSA patterns while providing updated, detailed forensic evidence from a contemporary Greek population.

The analysis of factors associated with disclosure timing revealed a few statistically significant determinants, suggesting that the mechanisms influencing CSA reporting are multifactorial but often subtle. Repeated victimization was strongly linked to delayed disclosure, confirming that chronic abuse fosters dependency, normalization of coercion, and fear of disbelief—processes repeatedly emphasized in international research [7]. In addition, the involvement of multiple medical specialties was significantly more common in delayed disclosures, likely to reflect greater diagnostic and psychosocial complexity among children and adolescents examined long after the incident [42]. Although not statistically significant, intrafamilial cases tended to be reported later, consistent with international evidence that relational proximity, dependence, and family secrecy suppress early reporting [6,28]. Conversely, the collection of genetic material was solely linked to early disclosures, highlighting the forensic consequences of delay. Overall, these findings suggest that repetition and relational dependency—rather than demographic characteristics—are the most influential determinants of delayed reporting in this cohort. Nevertheless, given the modest sample size, the study may have lacked sufficient statistical power to detect subtler associations, such as those related to family context or victim characteristics.

Recurrent abuse not only prolongs victimization but also intensifies psychological harm, creating a cycle of silence and re-victimization. Repeated exposure to sexual coercion can normalize abusive dynamics and foster learned helplessness and fear of disbelief [7]. When the perpetrator is a caregiver, the child may experience betrayal trauma—a dual violation of bodily integrity and emotional trust that can result in dissociation and memory fragmentation [43]. A significant proportion of victims in comparable cohorts have developed psychiatric symptoms—mainly phobic, anxious, or post-traumatic [11,44]. Meta-analytic data confirm that CSA significantly increases the risk of post-traumatic stress disorder, depression, suicidal ideation, and relational difficulties [44,45,46]. These outcomes are often more severe when the abuse is chronic or intrafamilial, where internalized guilt, maladaptive coping, and delayed disclosure reinforce secrecy and self-blame [8,47].

From a procedural standpoint, the findings reaffirm the importance of conducting forensic examinations under optimal conditions, by trained professionals, and as early as possible after the alleged assault [13]. Evidence collection is most effective within approximately 24 h in prepubertal children and up to 72 h in adolescents, maximizing the likelihood of detecting acute injuries and recovering biological traces. When these intervals have elapsed, examination should still be performed promptly, with emphasis on meticulous clinical assessment, detailed documentation of residual or healing lesions, and evaluation for possible chronic or recurrent abuse.

A trauma-informed, child-centred approach remains essential throughout the forensic process. Examinations should minimize distress, preserve dignity, and maintain psychological safety. Specialized pediatric forensic settings integrating medical, psychosocial, and legal expertise—such as the *Barnahus* or “Children’s House” model—represent international best practice. Originating in Iceland and adopted across Northern Europe, the Barnahus framework promotes interagency collaboration in child-friendly environments, minimizing secondary trauma [48,49]. Recent European developments also highlight the value of structured and standardized forensic workflows. For example, the accredited clinical forensic examination framework established at the Children’s Advocacy Center in Copenhagen (Justesen et al., 2025) demonstrates how cross-sectoral collaboration, systematic monitoring, and uniform documentation procedures can strengthen the quality of forensic evaluations and enhance procedural fairness [50]. In France, this multi-disciplinary approach has been adapted to a hospital context through the creation of Unités d’Accueil Pédiatrique Enfant en Danger (UAPED), formalized by the 2021 ministerial instruction (DGOS/R4/R3/R2/2021/220) [51]. These units integrate pediatric, psychological, and forensic expertise under medical coordination, ensuring comprehensive assessment and judicial reporting within a protected environment [31]. In Greece, the absence of such structured multidisciplinary protocols remains a major gap. Adapting the hospital-based French framework could enhance coordination, ensure timely referrals, and embed trauma-informed practice—an ethical and institutional imperative to protect and restore child survivors of sexual violence.

Certain limitations need to be acknowledged when interpreting the findings of this study. The relatively small sample size restricts the statistical power and precludes the detection of subtle associations between variables. Data were derived exclusively from medico-legal case files, which include information obtained through police referrals and forensic examinations. No laboratory results concerning biological material were available, as such analyses are performed and retained by the investigative authorities rather than the forensic service. Likewise, although child and adolescent psychiatric assessments may have been conducted in some cases, their findings were not accessible to the forensic doctor, reflecting the current lack of coordinated, multidisciplinary integration in Greece. Given the legal process in Greece, the reports from each appointed expert (medical, psychiatric, laboratory, or investigative) are submitted separately to judicial authorities, who then compile the data themselves. This fragmented process limits the holistic interpretation of each case. Μissing data in certain variables may have influenced the completeness of the analyses. Because of the pattern and scale of missingness, we did not apply imputation, and these variables were excluded from multivariate analyses. Consequently, the findings should be interpreted with caution, particularly in relation to predictors that were only sparsely reported. However, such gaps are inherent to medico-legal investigations of CSA cases, where disclosure and documentation are often limited by the sensitive nature of the events. Finally, all examinations were conducted and signed by a single certified forensic doctor. While this ensured uniformity in examination techniques and documentation practices, it may limit the generalizability of certain findings to other forensic settings or examiners. Although all examinations were conducted by a two-physician team (a certified forensic specialist and a resident), this procedure does not constitute a formal second independent expert opinion as implemented in certain countries where two specialists co-sign the report. This structural difference may limit the direct comparability of our forensic workflow with systems employing mandatory dual-expert assessments.

## 5. Conclusions

This study provides one of the few forensic analyses of child sexual abuse in Greece, contributing to the limited Southern European evidence base. The findings reveal patterns consistent with international data: most victims were female, and the mean age clustered around late childhood to early adolescence. The majority of perpetrators were adult males, and intrafamilial or recurrent abuse predominated. Delayed disclosure was common, significantly reducing the likelihood of detecting physical or biological evidence and underscoring that a normal examination does not exclude abuse. These observations emphasize the need for early, trauma-informed forensic assessment conducted by trained professionals within the first hours following disclosure.

At a systemic level, the study highlights the fragmented nature of the Greek response to child sexual abuse, where medical and investigative processes operate in parallel rather than collaboratively. Experience from European hospital-based multidisciplinary units shows that coordinated, child-centred structures can improve forensic documentation, reduce secondary trauma, and support timely clinical assessment. Developing similar integrated pathways in Greece could meaningfully strengthen both forensic practice and overall case management.

## Figures and Tables

**Table 1:** Descriptive statistics of the study variables (N = 89).

Variable	*n* (%), M ± SD
Age of victim	9.84 ± 4.53
Year	
2014–2019	37 (41.6)
2020–2024	52 (58.4)
Nationality	
Greek	66 (74.2)
Other	22 (24.7)
N/A	1 (1.1)
Sex of the victim	
Male	27 (30.3)
Female	62 (69.7)
Perpetrator	
Adult	28 (31.5)
Minor	12 (13.5)
N/A	49 (55.0)
Nationality of perpetrator	
Greek	50 (56.2)
Other	13 (14.6)
N/A	26 (29.2)
Gender of perpetrator	
Male	77 (86.5)
Female	2 (2.2)
Other	3 (3.4)
N/A	7 (7.8)
Relationship between victim and perpetrator	
Biological parent	36 (40.4)
Step-parent	4 (4.5)
Close relative/sibling	7 (7.9)
Friend of the family	6 (6.7)
School personnel	2 (2.2)
Stranger	4 (4.5)
Child or adolescent	7 (7.9)
N/A	23 (25.9)
Relationship between victim and perpetrator	
Intrafamilial	48 (53.9)
Extra-familial	18 (20.2)
N/A	23 (25.9)
Place of incident	
Home	44 (49.4)
School	5 (5.6)
Other	23 (25.8)
Ν/A	17 (19.1)
Times of child abuse	
1	18 (20.2)
2–5	5 (5.6)
>5	33 (37.1)
N/A	33 (37.1)
Number of perpetrators	
1	67 (75.3)
2	8 (9.0)
3	2 (2.2)
7	1 (1.1)
Multiple	2 (2.2)
N/A	9 (10.1)
Performance of toxicological analysis	
No	82 (93.2)
Yes	6 (6.8)
Type of alleged sexual act	
Sexual contact, touching	48 (53.9)
Intercourse	37 (41.6)
N/A	4 (4.5)
Means of sexual act	
Penis	20 (22.5)
Hands/fingers	16 (18.0)
Multiple	5 (5.6)
Ν/A	48 (53.9)
Physical injuries	
No	68 (76.4)
Yes	21 (23.6)
Findings from genitals	
No	73 (83.0)
Yes	15 (17.0)
Findings from anus	
No	85 (96.6)
Yes	3 (3.4)
Concurrent health problem	
No	55 (84.6)
Yes	10 (15.4)
Other specialty involved	
No	30 (34.9)
Yes	56 (65.1)
Time interval between abuse onset and first disclosure to authority (in days)	79.41 ± 130.79
Till 7 days	26 (29.2)
8–30 days	17 (19.1)
>30 days	17 (19.1)
N/A	29 (32.6)
Collection of genetic material	
No	71 (82.6)
Yes	15 (17.4)

*Notes*. Values refer to absolute and relative frequencies (%) or mean ± standard deviation (SD). N/A: not available.

**Table 2:** Crosstabulation between time taken to disclose and the study variables (N = 89).

Variable	Till 7 Days	8–30 Days	>30 Days	*p*
Year				
2014–2019	14 (53.8%)	6 (35.3%)	6 (35.3%)	0.356 *
2020–2024	12 (46.2%)	11 (64.7%)	11 (64.7%)	
Nationality				
Greek	17 (65.4%)	12 (70.6%)	13 (81.3%)	0.196 †
Other	9 (34.6%)	5 (29.4%)	3 (18.8%)	
Sex of the victim				
Male	7 (26.9%)	5 (29.4%)	7 (41.2%)	0.600 *
Female	19 (73.1%)	12 (70.6%)	10 (58.8%)	
Perpetrator				
Adult	12 (75.0%)	6 (54.5%)	4 (80.0%)	0.515 †
Minor	4 (25.0%)	5 (45.5%)	1 (20.0%)	
Nationality of perpetrator				
Greek	12 (66.7%)	9 (64.3%)	10 (83.3%)	0.504 †
Other	6 (33.3%)	5 (35.7%)	2 (16.7%)	
Gender of perpetrator				
Male	24 (96.0%)	16 (100.0%)	12 (100.0%)	>0.999 †
Female	1 (4.0%)	0 (0.0%)	0 (0.0%)	
Relationship between victim and perpetrator				
Intrafamilial	9 (56.3%)	10 (58.8%)	11 (91.7%)	0.099 *
Extra-familial	7 (43.8%)	7 (41.2%)	1 (8.3%)	
Place of incident				
Home	7 (33.3%)	10 (58.8%)	9 (69.2%)	0.214 †
School	2 (9.5%)	2 (11.8%)	0 (0.0%)	
Other	12 (57.1%)	5 (29.4%)	4 (30.8%)	
Times of child abuse				
1	13 (65.0%)	2 (14.3%)	3 (23.1%)	**0.006** †
2–5	3 (15.0%)	1 (7.1%)	1 (7.7%)	
>5	4 (20.0%)	11 (78.6%)	9 (69.2%)	
Number of perpetrators				
1	22 (84.6%)	15 (88.2%)	12 (80.0%)	0.446 †
2	4 (15.4%)	1 (5.9%)	2 (13.3%)	
3	0 (0.0%)	0 (0.0%)	1 (6.7%)	
7	0 (0.0%)	1 (5.9%)	0 (0.0%)	
Performance of toxicological analysis				
No	23 (88.5%)	17 (100.0%)	14 (87.5%)	0.439 †
Yes	3 (11.5%)	0 (0.0%)	2 (12.5%)	
Type of alleged sexual act				
Sexual contact, touching	12 (48.0%)	9 (56.3%)	10 (62.5%)	0.651 *
Intercourse	13 (52.0%)	7 (43.8%)	6 (37.5%)	
Means of perpetration				
Penis	8 (66.7%)	4 (40.0%)	3 (30.0%)	0.290 †
Hands/fingers	4 (33.3%)	4 (40.0%)	6 (60.0%)	
Multiple	0 (0.0%)	2 (20.0%)	1 (10.0%)	
Physical injuries				
No	19 (73.1%)	13 (76.5%)	14 (82.4%)	0.922 †
Yes	7 (26.9%)	4 (23.5%)	3 (17.6%)	
Findings from the genitals				
No	19 (76.0%)	13 (76.5%)	15 (88.2%)	0.715 †
Yes	6 (24.0%)	4 (23.5%)	2 (11.8%)	
Concurrent health problem				
No	20 (95.2%)	10 (83.3%)	12 (85.7%)	0.583 †
Yes	1 (4.8%)	2 (16.7%)	2 (14.3%)	
Other specialty involved				
No	16 (61.5%)	4 (26.7%)	5 (31.3%)	0.047 *
Yes	10 (38.5%)	11 (73.3%)	11 (68.8%)	
Collection of genetic material				
No	12 (48.0%)	16 (94.1%)	17 (100.0%)	**<0.001** †
Yes	14 (53.8%)	0 (0.0%)	0 (0.0%)	

*Notes*. Values refer to absolute and relative frequencies (%). * chi-square test, † Monte Carlo simulation test.

## Data Availability

Data supporting reported results are available upon reasonable request from the corresponding author. The data are not publicly available due to privacy restrictions.
